# 3.C. Workshop: The WHO European Health Report 2021: overcoming gaps in data and health information systems

**DOI:** 10.1093/eurpub/ckac129.130

**Published:** 2022-10-25

**Authors:** Organised by: WHO Europe, Chair persons: Natasha Azzopardi Muscat (WHO Europe)

## Abstract

The publication of the European Health Report every three years gives readers a vital snapshot of health in the WHO European Region and progress towards health and well-being for all. The European Health Report 2021 (EHR2021) shows trends in and progress towards the health-related Sustainable Development Goals (SDGs) and the goals of the European Programme of Work 2020-2025 (EPW). It reveals gaps in progress, persistent health inequalities and other areas of concern and uncertainty, where action must be taken. The Report also lays bare, and not for the first time, considerable data gaps for key indicators, as well as operational problems in Member States’ (MS) health information systems (HISs). As strong HIS are a prerequisite for evidence-informed policy-making, overcoming these issues is critical. In this workshop, we will first present the main findings of the EHR2021, showing which challenges the European Region is facing related to the three core priorities of the EPW: moving towards universal health coverage (UHC), protecting people better against health emergencies and ensuring healthy lives and well-being. Although from the EHR2021 it shows that data for many SDG indicators can be reported, there are also many areas that we cannot monitor well due a lack of data. Such data gaps are not stand-alone problems but relate to wider issues with the functioning of HISs. In the second presentation we will present an overview of the most important data gaps for key indicators and common issues in national HISs in the European Region, providing insight into where concretely action needs to be taken to ensure a stronger evidence-base for policy-making. Supporting MS in strengthening their HIS has traditionally been an important focus in WHO Regional Office for Europe's (WHO-Euro's) work and continues to be so under the EPW. One important tool that WHO-Euro uses for this purpose is a HIS assessment. Such an assessment provides insight into the strengths and weaknesses of the HIS and is the starting point for an evidence-informed process of HIS strengthening. In the third presentation, we will present WHO-Euro's HIS assessment approach. Lacking or incomplete digitalisation is a common issue in HISs in the Region, and the COVID-19 pandemic has emphasized the need for implementing digital solutions to improve the efficiency of HISs. Therefore, supporting Member States in the further digitalisation of their HIS is an important focus of WHO-Euros HIS strengthening work for the coming years. In the final presentation, we will present WHO-Euro's existing tools and planned activities related to this. Participants of this workshop will get a comprehensive overview of current challenges related to achieving the health-related SDGs in Europe, with a specific focus on challenges related to health information. They will learn about WHO-Euro's approaches and plans for how to tackle these challenges.

**Key messages:**

• Although the European Health Report 2021 shows data for many health-related SDGs, there are important areas that we cannot monitor well due to a lack of data and issues in health information systems.

• Overcoming these issues is crucial for a strong evidence-base for health policy, and therefore WHO Regional Office for Europe has a series of tools and activities to support Member States in this.

